# Information extraction from weakly structured radiological reports with natural language queries

**DOI:** 10.1007/s00330-023-09977-3

**Published:** 2023-07-28

**Authors:** Amin Dada, Tim Leon Ufer, Moon Kim, Max Hasin, Nicola Spieker, Michael Forsting, Felix Nensa, Jan Egger, Jens Kleesiek

**Affiliations:** 1 Institute of AI in Medicine (IKIM), University Hospital Essen, Girardetstraße 2, 45131 Essen, Germany; 2 Dr. Krüger MVZ GmbH, Bocholt, Germany; 3 Institute of Diagnostic and Interventional Radiology and Neuroradiology, University Hospital Essen, Essen, Germany; 4 Cancer Research Center Cologne Essen (CCCE), University Medicine Essen, Essen, Germany; 5 German Cancer Consortium (DKTK), Partner Site Essen, Essen, Germany

**Keywords:** Information extraction, Natural language processing, Machine learning

## Abstract

**Objectives:**

Provide physicians and researchers an efficient way to extract information from weakly structured radiology reports with natural language processing (NLP) machine learning models.

**Methods:**

We evaluate seven different German bidirectional encoder representations from transformers (BERT) models on a dataset of 857,783 unlabeled radiology reports and an annotated reading comprehension dataset in the format of SQuAD 2.0 based on 1223 additional reports.

**Results:**

Continued pre-training of a BERT model on the radiology dataset and a medical online encyclopedia resulted in the most accurate model with an F1-score of 83.97% and an exact match score of 71.63% for answerable questions and 96.01% accuracy in detecting unanswerable questions. Fine-tuning a non-medical model without further pre-training led to the lowest-performing model. The final model proved stable against variation in the formulations of questions and in dealing with questions on topics excluded from the training set.

**Conclusions:**

General domain BERT models further pre-trained on radiological data achieve high accuracy in answering questions on radiology reports. We propose to integrate our approach into the workflow of medical practitioners and researchers to extract information from radiology reports.

**Clinical relevance statement:**

By reducing the need for manual searches of radiology reports, radiologists’ resources are freed up, which indirectly benefits patients.

**Key Points:**

*• BERT models pre-trained on general domain datasets and radiology reports achieve high accuracy (83.97% F1-score) on question-answering for radiology reports.*

*• The best performing model achieves an F1-score of 83.97% for answerable questions and 96.01% accuracy for questions without an answer.*

*• Additional radiology-specific pretraining of all investigated BERT models improves their performance.*

**Graphical Abstract:**

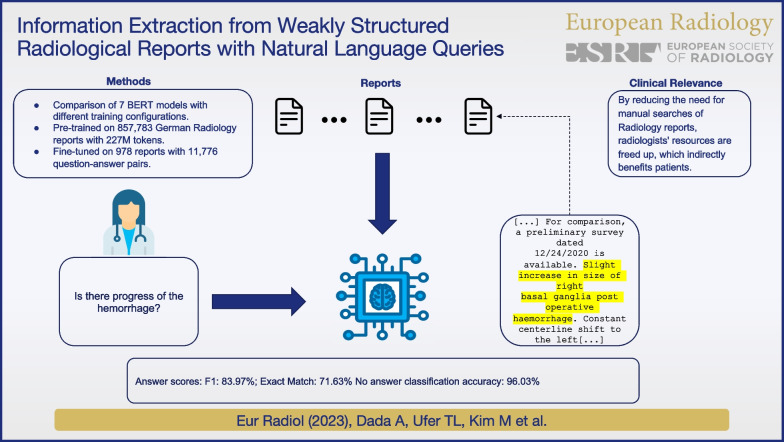

**Supplementary Information:**

The online version contains supplementary material available at 10.1007/s00330-023-09977-3.

## Introduction

Radiology reports significantly impact clinical decision-making. Therefore, they have to be prepared with utmost care. An elementary component of evaluating radiology imaging is the comparison of the latest findings with past findings. Commonly, only the dynamics of a finding over time allow for a reliable interpretation. For instance, dependent on an increase or decrease in size, the treatment of a lesion varies. Often enough, the comparison with past radiology reports requires significant effort. This is attributable to the fact that radiology reports are weakly structured.

Every radiologist writes in their style with the consequence that it is challenging to compare the reports from various radiologists directly, not only regarding their structure but also regarding their choice of words to describe a particular finding. On the other hand, the information density in radiology reports is high to such an extent that it is inadequate to skim the text. Practitioners must read the reports carefully to capture every critical piece of information. Additionally, radiology reports written in prose aggravate the extraction of relevant information. Integrating past reports strains the already scarce resources of radiology specialists, and it is even more true for medically challenging cases where it becomes necessary to review multiple previous reports. Furthermore, the difficulty in accessing information is particularly great for radiologists from different institutions, referring physicians, and non-physician practitioners.

Despite attempts to introduce more structure in radiology reports through templates (e.g., for BiRADS classifications), a substantial part of a template is still formulated in free text. Moreover, even if future reports were more structured, renouncing past reports’ information would be a significant drawback. Therefore, holding an application that automatically shows the desired passage in all past reports on request would be valuable.

In recent years the field of natural language processing (NLP) shifted toward deep learning methods [1]. One of these methods is attention-based transformer models, particularly bidirectional encoder representations from transformers (BERT) [2]. In contrast to prior approaches, they can be trained on large volumes of unlabeled data and then refined on a relatively small labeled dataset. Bressem et al [3] evaluated two BERT models pre-trained on general domain data and two trained on 3.8M radiology reports. The models were trained to detect nine findings (e.g., congestion, gastric tube, thoracic drains) on 5203 annotated radiology reports. Datta et al [4] focused on extracting spatial information from radiology reports by applying a BERT model in two steps. First is the recognition of spatial triggers. Second is determining the relationship between these triggers. They trained the models on a dataset of 400 manually annotated radiology reports. Wen et al [5] trained a BERT reading comprehension question answering (RCQA) model on the publicly available emrQA dataset [6] which consists of electronic medical records. Additionally, they complement the training with i2b2 notes^1^ from their institution and samples from SQUAD 2.0 [7]. They limited their set of questions to sentences that start with the word “why.” Radiology reports are usually divided into a finding and interpretation section. Recent studies by Liang et al [8] and Fink et al [9] showed that BERT models perform similarly well to experts in terms of correctness and comprehensibility when deriving tumor progression based on finding sections. Overall, transformers in radiology have clearly outperformed other NLP methods across different languages in recent years [9–12].

Inspired by these studies, our research aims at evaluating the performance of German BERT models pre-trained on radiology reports for information extraction via question answering. We utilize 857,783 available reports and a previously released pretrained German BERT model to establish multiple radiological BERT models and fine-tune them to an RCQA dataset with 1223 reports annotated in our institution with questions formulated by trained medical staff and medical students.

A limitation of the methodology proposed by Bressem et al [3] is its representation of the problem as a classification task, constraining the model’s abilities to a predetermined set of 9 categories. Additionally, their model does not yield the position of information within a note, further restricting its general applicability. Similarly, Datta et al [4] employed a named entity recognition (NER) approach to determine the exact positions of entities within a text. However, their method is also limited to their predefined set of entity labels. Wen et al [5] proposed an RCQA approach that addresses the limitations of predefined classes and missing spatial information. They trained on the emrQA dataset which is constructed by automatic generation using templates for questions that can be answered using existing annotations from the i2b2 datasets. Nonetheless, their approach is still constrained to the NER classes defined in the source datasets. Furthermore, their questions are limited to phrases starting with “why,” which restricts the set of possible questions.

In contrast, our approach is based on a RCQA dataset, overcoming the limitation of having a fixed number of classification or NER labels. We do not rely on NER datasets, as we manually annotate question-answer pairs instead of generating the dataset from existing ones. Finally, we formulate our set of questions based on the perspectives of radiologists, ensuring the applicability of our approach to a clinical setting.

## Material and methods

This work uses two types of datasets. First are datasets that consist of plain text without annotations for the unsupervised pre-training of the BERT models. Second is a manually annotated fine-tuning dataset consisting of question-answer pairs from radiological reports.

### Pre-training data

We built the dataset from reports collected retrospectively from the radiological information system of Essen University Hospital. We gathered 857,783 reports with 92M words and 227M tokens written between 23.08.1999 and 17.06.2021, covering most major CT and MRI modalities (see Table A.1 in the supplementary material). We randomly shuffle and merge the reports into a single text file with a size of 781 MB to pre-train the transformer models. The ratio between CT and MRI reports is approximately 70%/30%.

As a complementary dataset, we obtain a dump of DocCheck Flexikon^2^, an open medical encyclopedia maintained by over 5000 authors, mainly composed of physicians and medical students. It consists of 14,825 articles across all medical specialties with 3.7M words and 7.6M tokens^3^.

### Fine-tuning data

As a basis for the RCQA dataset, we collected 1223 additional radiology reports limited to brain CT scans. Three medical student assistants in their sixth and eighth semesters annotated 29,273 question-answer pairs. To prevent overlap between pre-training and fine-tuning data, we collected reports written after 17.06.2021. Due to its popularity in past publications, the dataset follows the SQuAD 2.0 format. The format allows comparability with other RCQA research and simple implementation because of its support by frameworks.

In contrast to SQuAD 2.0, we provide the annotators with a list of questions we define with the help of a radiologist. We made this decision based on two points. First, the diversity of the original SQuAD 2.0 dataset requires different questions for different articles. For example, questions about historical events are very different from questions about chemical elements. Conversely, a radiologist’s questions are limited to specific findings (e.g., the progression of tumors). Second, we must consider our limited human and time resources. The annotation of SQuAD 2.0 was crowdsourced, which is not an option for sensitive and challenging clinical data. To evaluate our models’ ability to answer different questions than the predefined ones, we asked the annotators to create one custom question for at least every third report.

We group the questions into categories based on common radiological observations (e.g., MRI signal changes). The supplementary material lists all questions and their corresponding categories and provides more details on the RCQA dataset.

### Models

In this study, we utilize two publicly available BERT models. The first (G-BERT) released by deepset [12] is trained on, a Wikipedia dump, OpenLegalData^4^, and news articles. We use the model uploaded to Hugging Face [13].

The second model (GM-BERT) [14] is G-BERT further pre-trained on German medical articles collected from various internet sources. The sources include the websites sprechzimmer^5^, netdoktor^6^, doktorweigl^7^, onmeda^8^, krank^9^, internisten-im-netz^10^, apothekenumschau^11^, and vitanet^12^. In total, the dataset consists of 194.5 MB of text.

#### Pre-trained models

We add a classification layer to the pre-trained G-BERT and GM-BERT models to predict for each report token the probability of being the start or end token of the answer span. The inputs to the model are concatenations of the question and the report of each sample. In addition to the span boundaries, it predicts a probability for the CLS token, which encodes sentence-level information for classification tasks. For questions without an answer, the model maps both the start and the end tokens to the CLS token.

The pre-training data of G-BERT only includes general domain data. While GM-BERT was further pre-trained on medical domain data, the data differs from radiological reports. We address this limitation by continuing the pre-training of both models on our radiological report dataset and the data we collected from Flexikon. We refer to these models as G-BERT+Rad, GM-BERT+Rad, G-BERT+Rad+Flex, and GM-BERT+Rad+Flex.

#### From scratch

Additionally, we initialized a RoBERTa [15] model due to its improved results on SQuAD. We refer to it as RadBERT. RoBERTa uses a byte-pair encoding (BPE) tokenizer with a vocabulary size of 50,000 tokens. Since our dataset is much smaller than the one used in the RoBERTa paper (160 GB vs. 781 MB), we decided to decrease the vocabulary size to 8000 tokens and reduce the number of hidden layers from 12 to 6, to avoid excessive computational complexity and overfitting.

The supplementary material contains a description of the training configuration of the models.

### Metrics

To evaluate the pre-training, we calculate the number of correctly predicted tokens for the masked language modeling (MLM). We do this once for the token with the highest scored token (HST) and once for the five highest scored tokens (5HST). Matches occur if the token with the highest prediction score, or one of the 5HST, matches the masked token. We then divide the number of correctly predicted tokens by the total number of masked tokens. We use the same metrics SQuAD uses Exact match (EM) and F1-score for the question-answering performance. EM measures the percentage of predictions that match the ground truth answers. The F1-score measures the average overlap between the prediction and the ground truth answer. True positives and negatives are tokens that the correct answer and the model prediction share or which both do not include. False positives include tokens the model prediction contains, but the correct answer does not. Whereas false negatives are tokens, the correct answer contains, but the model prediction does not.

In contrast to SQuAD, we exclude questions without an answer from the F1 and EM scores since we noticed that the models were significantly better in classifying unanswerable questions than in determining the position of an answer. This led to an overestimation of the actual model score. To address this, we measure the binary classification task of identifying unanswerable questions with a separate accuracy score.

## Results

We evaluated the pre-trained models on a subset of 1000 sentences from radiology reports we excluded from the training set. We randomly mask a token for each sentence and let the models predict this missing token. This process is repeated five times for each model. Table 1 presents the resulting average, variance, and standard deviation of the HST and 5HST scores we observed during this evaluation.

**Table 1:** Results of the pre-training evaluation

Models	Highest scored token			5 highest scored tokens		
	Average	Variance	Standard dev.	Average	Variance	Standard dev.
G-BERT	14.4%	0.00%	0.6%	23.3%	0.01%	0.98%
G-BERT+Rad	37.33%	0.01%	0.81%	42.94%	0.01%	1.07%
G-BERT+Rad+Flex	35.11%	0.01%	0.01%	40.56%	0.01%	0.83%
GM-BERT	14.11%	0.01%	0.86%	23.74%	0.00%	0.7%
GM-BERT+Rad	37.02%	0.02%	1.29%	42.72%	0.01%	1.17%
GM-BERT+Rad+Flex	34.61%	0.01%	1.13%	40.53%	0.00%	0.63%
RadBERT	**48.05%**	0.00%	0.73%	**66.46%**	0.00%	0.94%

RadBERT outperforms all other models with an HST accuracy of 48.05% and 5HST of 66.46%. The lowest-performing models are G-BERT and GM-BERT, with HST accuracies of 14.4%/14.11% and 5HST scores of 23.3%/23.74%. The lowest-performing models were never trained on radiology reports, while RadBERT was solely trained on radiological data. The additional pre-training on medical data of GM-BERT results in no significant difference from G-BERT. The models that were further pre-trained on radiological reports indicate a significant boost in performance (+22.93% HST for G-BERT and +22.91% for GM-BERT). However, both models still achieve lower scores than RadBERT. The additional pre-training on our Flexicon dataset decreased the MLM performance of both models.

### Fine-tuning results

Table 2 displays the mean EM and F1-score across all five validation folds for questions that can be answered. The F1-score of G-BERT+Rad+Flex (83.97%) and the EM-score of GM-BERT+Rad+Flex (71.81%) are the highest scores in the RCQA task evaluation. G-BERT and GM-BERT achieve the lowest precision, while all other models benefit from the pre-training on our custom datasets. Without further pre-training, GM-BERT performs better than G-BERT. Except for RadBERT, all models reach their peak performance after the first training epoch. Afterward, their performance decreases, indicating overfitting. Therefore, we implicitly apply early stopping by saving the model state after each epoch and finally loading the best-performing state. Conversely, RadBERT improves up until the fifth epoch. Therefore, we decided to continue its training for another five epochs, leading to an improvement of +0.51% F1 and +1.43% EM. In contrast to the other models, RadBERT converges slower to lower scores.

**Table 2:** Precision of fine-tuned models on answerable questions

Model	EM	F1
G-BERT	28.73%	35.89%
G-BERT+Rad	70.04%	83.33%
G-BERT+Rad+Flex	71.63%	**83.97%**
GM-BERT	35.06%	45.86%
GM-BERT+Rad	70.22%	83.12%
GM-BERT+Rad+Flex	**71.81%**	83.80%
RadBERT	55.93%	70.26%

The classification accuracy of unanswerable questions is presented in Table 3. In this evaluation, GM-BERT+Rad+Flex achieved the highest accuracy. Additionally, G-BERT and GM-BERT performed better than the model trained from scratch. The ranking of the other models remained similar to the results for answerable questions. However, the overall accuracy is significantly higher.

**Table 3:** Precision of fine-tuned models on unanswerable questions

Model	EM
G-BERT	89.59%
G-BERT+Rad	95.52%
G-BERT+Rad+Flex	96.01%
GM-BERT	89.97%
GM-BERT+Rad	95.32%
GM-BERT+Rad+Flex	**96.12%**
RadBERT	87.37%

The pre-training dataset has significantly more CT reports than MRI reports. We performed an additional evaluation to investigate this imbalance’s impact on the models pre-trained on our radiology dataset. As shown in Table 4, we observe that all models have a slight advantage on CT reports.

**Table 4:** Precision of fine-tuned models on answerable questions in CT vs. MRI reports

	CT		MRI	
Category	F1	EM	F1	EM
G-BERT+Rad	84.15%	70.94%	81.11%	69.10%
G-BERT+Rad+Flex	85.43%	72.40%	81.28%	69.84%
GM-BERT+Rad	84.12%	71.09%	80.66%	69.04%
GM-BERT+Rad+Flex	85.01%	72.48%	81.29%	70.01%
RadBERT	71.78%	56.88%	69.62%	56.34%

### Category-wise evaluation

One of our motivations for using a question-answering model was its potential capability to generalize across unseen questions. In contrast to classification models, it learns the mapping between categories and text passages implicitly through question-answer pairs instead of explicit classification labels. Therefore, it could deal with examples from unseen categories. We explore this assumption for answerable questions with G-BERT+Rad+Flex, the model that achieved the highest F1-score in the evaluation.

We first compute the evaluation scores for each category separately. Afterward, we create an individual training set for each category by excluding the category from the training set. We train G-BERT+Rad+Flex on each training set that is missing one category. These models are then evaluated on the category they have not seen during the training. Table 5 displays the results of the category-wise evaluation and the corresponding results of the models that have not seen the category they are evaluated against.

**Table 5:** Results of the category-wise evaluation on the entire dataset and subsets without the respective category. The custom category refers to the questions the annotators formulated during the annotation process

	Category-wise		Leave-one-out	
Category	F1	EM	F1	EM
Extraneous material	94.12%	85.52%	93.27%	85.94%
MRI signal changes	72.58%	48.65%	68.30%	46.44%
Oncology	71.09%	53.03%	67.34%	51.44%
Hemorrhage	83.38%	63.81%	80.20%	63.16%
Ischemia	65.48%	52.80%	69.13%	55.83%
Inflammation	60.04%	39.41%	60.24%	40.02%
CSF circulation	94.71%	90.00%	95.11%	90.46%
Edema	76.12%	66.30%	71.90%	62.81%
Custom	78.33%	62.01%	-	-

The results for the categories extraneous material, inflammation, and CSF circulation are relatively similar, indicating that the model can deal with these questions without prior training on them. The evaluation of MRI signal changes, oncology, hemorrhage, and edema displays the expected drop in accuracy due to the missing training examples. However, the drop is relatively slight. These observations indicate a good generalization over unknown question-answer pairs. Surprisingly, we observed an increased performance on the category ischemia for the model that was not trained on it. This may relate to the relatively low number of examples from this category and overfitting to more frequent categories. For the custom questions the annotators formulated, we report an F1-score of 78.33% and an EM score of 62.01%. These results are close to the scores across all categories (83.97% F1-score and 71.63% EM score), supporting our claim that the model can answer unseen questions.

## Discussion

Our experiments show that further radiology-specific pre-training of a transformer model trained on general domain data results in the highest precision for radiology RCQA. Conversely, a model without medical domain pre-training shows the lowest precision. Additionally, further medical pre-training of available models results in better models than training a model from scratch. One possible explanation is that a model trained from scratch cannot develop sufficient language comprehension due to the similarity of the texts. Although RadBERT achieves the highest MLM scores, the other models benefit from their extensive training on natural language leading to a better question-answering capability. Another difference is the size of the pre-training data (781 MB for RadBERT vs. 12 GB for G-BERT). An extensive dataset is crucial for training BERT models [16].

G-BERT and GM-BERT have shown similar evaluation scores. We assume that the continued pre-training on 194.5 MB of medical articles GM-BERT received is not enough to change the weights of G-BERT significantly, or the articles used are too broadly scattered in the medical domain to improve radiology report comprehension. Articles used for further pre-training GM-BERT may be too close to texts used in the original G-BERT, like medical Wikipedia articles, to influence the weights. This finding is also indicated by the increase in performance after training on 781 MB radiology reports, showing that an increased performance through training on medical data is possible.

The evaluation of the custom formulated questions and the training runs that excluded one question category indicated generalization over unseen questions. Therefore, we think the model is stable against variations in the formulation of questions, deviating medical language used by various medical practitioners, reports on other anatomical regions than the head, and different imaging modalities. Generalization is only possible to a certain degree, for instance, due to vocabulary that is not included in the training set.

We were able to show that our model generalizes to unseen questions. This is a clear advantage over previously introduced classification and NER models. The only other application of a BERT model for medical RCQA we are aware of was reported by Wen et al [5]. In contrast to their study, we did not restrict our dataset to why-questions. Although our best model achieves a higher accuracy for answerable questions (84.0% F1-score vs. 73.5% and 71.6% EM score vs. 67.2%), a direct comparison is unfeasible due to the different datasets. Wen et al trained on English clinical notes compared to our German radiology dataset.

In this work, we have dealt exclusively with German models. However, the approach can be applied to any language for which pre-trained transformer models are available. Especially in English there are models already pre-trained on clinical data [17, 18].

One important limitation of our approach is the restriction of answers to a single, consecutive text span. In practice, however, the answer might consist of multiple spans located at different locations throughout the text or across multiple texts. This can either be because some parts only answer the question partially or because there are several valid, possibly contradicting, answers to a question. In future research, we want to address this limitation with multiple span models based on previous approaches (e.g., [19–21]). Additionally, the interpretability of transformers remains an open question. We think that future research on interpretability opens up opportunities to discover weaknesses in the models’ reasoning and can build trust to incorporate the models into practical settings.

We explored different approaches to infuse BERT models with radiology knowledge to establish models with reading comprehension for reports. The models have shown high precision and evidence of good generalization. Our approach can be transferred to other downstream tasks and medical fields with little effort to provide medical professionals and researchers with a powerful tool to process large amounts of text without developing new algorithms for each task.

### Supplementary Information

Below is the link to the electronic supplementary material.

Supplementary file1 (PDF 117 KB)

## Abbreviations

BERT: Bidirectional encoder representations from transformers
BPE: Byte-pair encoding
EM: Exact match
HST: Highest scored token
MLM: Masked language modeling
NLP: Natural language processing
RCQA: Reading comprehension question answering

## Footnotes

1 https://www.i2b2.org/NLP/DataSets/Main.php

2 https://flexikon.doccheck.com/

3 Applying the tokenizer of deepset’s base model [12].

4 https://de.openlegaldata.io/

5 www.sprechzimmer.ch

6 www.netdoktor.de

7 www.doktorweigl.de

8 www.onmeda.de

9 www.krank.de

10 www.internisten-im-netz.de

11 www.apotheken-umschau.de

12 www.vitanet.de

## Declarations

### Guarantor

The scientific guarantor of this publication is Prof. Jens Kleesiek.

### Statistics and biometry

No complex statistical methods were necessary for this paper.

### Methodology

• retrospective

• technical study

• performed at one institution
