# Supplementary material for: Information extraction from weakly structured radiological reports with natural language queries
Source: Eur Radiol. 2023 Jul 28;34(1):330–7. doi: 10.1007/s00330-023-09977-3 (PMC10791738; doi:10.1007/s00330-023-09977-3)
Supplement: Supplementary file 1 — Supplementary file1 (PDF 117 KB) [file 330_2023_9977_MOESM1_ESM.pdf]

Information extraction from weakly structured radiological reports with natural language queries

Electronic Supplementary Material

Training Configuration

We continued the pre-training of G-BERT and GM-BERT for two epochs on each dataset with the Adam optimizer. We configured a learning rate of 5e-5 with 500 warm-up steps and a weight decay of 0.01. The maximal sequence length of inputs is 512 tokens. We split reports that exceed this range into smaller chunks that we process individually. The stride length between these smaller subdivisions is 128 tokens. RadBERT was trained with the AdamW for ten epochs, while all other parameters are the same as for the other models. We used a batch size of 12 for G-BERT and GM-BERT and a batch size of 16 for RadBERT.

RCQA Dataset

Table A.3 in the supplementary material lists all questions and their corresponding categories. We collected very few answers for some questions since some observations occur less frequently. This prevented us from making reliable statements on the capabilities of our models regarding these questions. To address this, we extended the dataset by performing a free-text search with a set of keywords that we assume to correlate with underrepresented questions (the supplementary material contains an overview of the keywords). We searched with regular expressions to exclude negated sentences and account for various word endings. We gathered 226 additional Head CT reports for the four rarest categories with this procedure. Figure A.2 shows the distribution of answerable questions after adding the new reports.

The annotation process resulted in about twice as many unanswerable (19,460) questions than answerable (9,813) ones since many predefined questions are not answerable. For instance, tumor-specific questions are not answerable in a report about a patient with a fracture. Following SQuAD 2.0, we deleted question-answer pairs to achieve a 2:1 ratio of answerable to unanswerable examples.

Afterward, we shuffle the reports and split them into five train and test folds based on the total number of reports (i.e., five splits with 80%/20% of the total reports). Each training fold contains 978 reports with an average number of 11,776 question-answer pairs. The test folds contain 245 reports and 3,078.4 question-answer pairs on average.

Figure A.1: Number of question-answer pairs for rarest questions. The numbering corresponds to the order of the questions in Table A.3

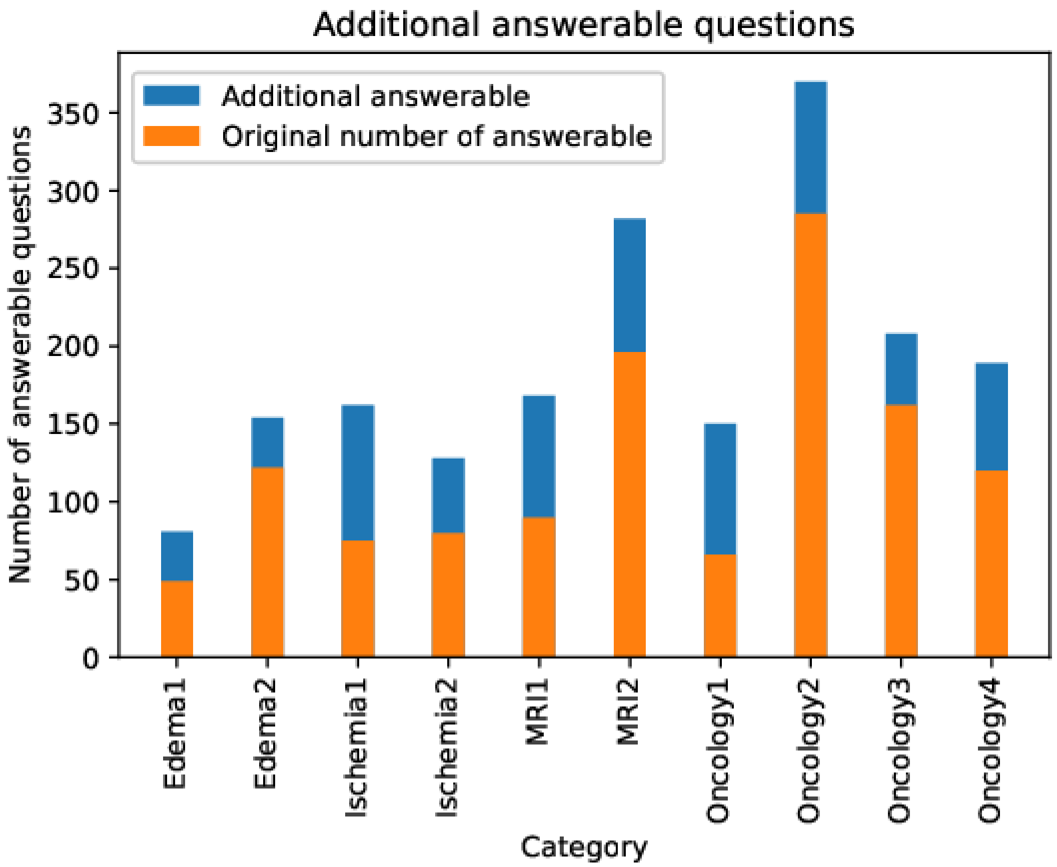

Table A.1: Type of modality and occurrence in the dataset.

Modalities	Number
CTAB	160321
CTB	9977
CTGE	9991
CTH	6312
CTOE	2976
CTS	170810
CTSH	28954
CTT	173826
CTTA	13236
CTUE	4280
CTWS	16242
MRA	17075
MRB	5305
MRBE	10833
MRH	12128
MRL	16357
MRM	10712
MROE	6120
MRS	143135
MRSH	9416
MRTH	3832
MRUE	14777
MRWB	3656
MRWS	7512

Table A.2: Categories and questions in the RCQA dataset.

Category	Question
Allgemein	Klinische Angaben?
	(Haupt-)Fragestellung?
	Welche Untersuchung?
	Liegt eine Voruntersuchung vor?
	Liegt eine akute Pathologie vor?
	Gibt es eine raumfordernde Komponente der akuten Pathologie (Mittellinienverschiebung/ Gefahr einer Einklemmung)?
	Wurde ein Arzt kontaktiert aufgrund der akuten Patholgie?
	Anatomische Lage des Hauptbefund?
	Ist der Hauptbefund unauffällig?
	Liegen Nebenbefunde vor?
Blutung	Liegt eine neue Blutung vor?
	Verlaufsbeurteilung der Blutung?
Fremdmaterial	Ist die Installation lagekorrekt?
Ischämie	Liegt eine neue Ischämie/Infarktdemarkation vor?
	Verlaufsbeurteilung der Ischämie/Infarktdemarkation?
Liquorzirkulation	Liegt eine neue Liquorzirkulationsstörung vor?
	Verlaufsbeurteilung der Ventrikelweite?
Ödem	Liegt ein neues Hirnödem vor?
	Verlaufsbeurteilung des Hirnödems?
Entzündung	Liegt eine neue Entzündung/Läsion vor?
	Verlaufsbeurteilung der Entzündung/Läsion?
MRT Signalveränderung	Liegt eine Diffusionsstörung vor?
	Liegt eine FLAIR-Hyperintensität vor?
Onkologie	Liegt eine neue Tumormanifestation vor?

	Verlaufsbeurteilung der Tumormanifestation?
	Beurteilung Perifokalödem
	Gibt es Nachweis von Metastasen?

Table A.3: Translation of the categories and questions in the RCQA dataset.

**Category**	**Question**
General	Clinical information?
	(Main) question?
	Which examination?
	Is there a prior examination?
	Is there an acute pathology?
	Does the acute pathology have a space-occupying component? (midline shift/ danger of entrapment)?
	Was a doctor contacted due to the acute pathology?
	Anatomical location of the main finding?
	Is the main finding inconspicuous?
	Are there secondary findings?
Hemorrhage	Is there a new hemorrhage?
	Progress of the hemorrhage?
Foreign material	Are the lines, tubes, and devices positioned correctly?
Ischemia	Is there new evidence of ischemia or infarct?
	Progression of ischemia or infarct?
CSF circulation	Is there a disturbance in CSF circulation?
	Progression of the ventricle width?
Edema	Is there new cerebral edema?

	Progression of the cerebral edema?
Inflammation	Is there a new inflammation/lesion?
	Progression of the inflammation/lesion?
MRI signal changes	Is there a diffusion disturbance?
	Is there a T2 hyperintensity?
Oncology	Is there a new tumor manifestation?
	Progression of the tumor manifestation?
	Is there perifocal edema?
	Is there evidence of metastases?

Table A.4: Search keywords for additional examples of less common categories.

**Category**	**Keywords**
Ischämie	['ischämie', 'infarktdemarkation', 'infarkt', 'mikroangiopathie']
Ödem	['ödem', 'schwellung', 'flüssigkeitsansammlungen', 'wasseransammlungen']
MRT Signalveränderung	['diffusionsstörung', 'diffusionsrestriktion', 'diffusionseingeschränkt', 'schrankengestört', 'schrankenstörung']
Onkologie	['tumor', 'meningeom', 'raumforderung', 'gliom', 'lymphom', 'meningeosis', 'meningeose', 'metastase', 'oligodendrogliom', 'osteom']

Table A.5: Translation of search keywords for additional examples of less common categories.

**Category**	**Keywords**
Ischemia	['ischemia', 'infarct demarcation', 'infarct', 'microangiopathy']
Edema	['edema', 'swelling', 'fluid accumulation', 'water accumulation']
MRI signal changes	['diffusion disturbance', 'restricted diffusion', 'diffusion restricted', 'disrupted blood-brain barrier']
Oncology	['tumor', 'meningioma', 'space occupying lesion', 'glioma', 'lymphoma', 'spread', 'metastasis', 'oligodendroglioma', 'osteoma']
